# Treatment of esophageal cancer: surgical outcomes of 335 cases operated in a single center

**DOI:** 10.1590/0100-6991e-20202723

**Published:** 2021-02-05

**Authors:** RAPHAELLA PAULA FERREIRA, DANILO SAAVEDRA BUSSYGUIN, HYGOR TROMBETTA, VICTOR JOSE DORNELAS MELO, DANIELE REZENDE XIMENEZ, VINICIUS BASSO PRETI, GERARDO CRISTINO GAVARRETE VALLADARES, FLAVIO DANIEL SAAVEDRA TOMASICH, PHILLIPE ABREU

**Affiliations:** 1 - Faculdade de Ciências Médicas da Santa Casa de São Paulo, Departamento de Cirurgia - São Paulo - SP - Brasil; 2 - Hospital Erasto Gaertner, Centro de Projetos de Ensino e Pesquisa - Curitiba - PR - Brasil; 3 - Hospital Erasto Gaertner, Departamento de Cirurgia, Serviço de Cirurgia Abdominal - Curitiba - PR - Brasil; 4 - University of Miami, Jackson Memorial Hospital, Department of Surgery - Miami - FL - EUA

**Keywords:** Esophageal Neoplasms, Esophagectomy, Postoperative Complications, Treatment Outcome, Neoplasias Esofágicas, Esofagectomia, Complicações Pós-Operatórias, Avaliação de Resultado de Intervenções Terapêuticas

## Abstract

**Objectives:**

the surgical approach persists as the main treatment for esophageal cancer. This study compares the patients of the same institution over time at three different times*.*

**Methods:**

this is a retrospective, observational, descriptive study comparing the surgical outcomes obtained by the Division of Surgical Oncology of Erasto Gaertner Hospital. The sample was divided into Period 1 (1987-1997), Period 2 (1998-2003) and Period 3 (2007-2015). Survival rates and disease-free survival were estimated by the Kaplan-Maier method. Survival predictors were identified with Cox regression. ANOVA test was used for comparison between groups. Data were analyzed with SPSS 25.0 and STATA 16, and p<0.05 was considered statistically significant*.*

**Results:**

a total of 335 patients underwent esophagectomy or esophagogastrectomy. When the clinical characteristics of the 3 groups were compared, there was no statistically significant difference. Neoadjuvance was significantly higher in Period 3 (55.4% of patients). We found a histological change in the diagnosis over time, with a significant increase in adenocarcinoma. Morbidity and mortality rates were higher in Period 3. The main complications were pulmonary and anastomotic fistulas. Overall survival in 5 years increased over time, reaching 59.7% in Period 3.

**Conclusions:**

better neoadjuvant treatment contributed to increase the global survival of patients, despite greater rate of immediate complications to surgery.

## RESUMO

**Objetivo:**

A abordagem cirúrgica persiste como tratamento principal para o câncer de esôfago. O presente estudo compara as casuísticas da mesma instituição ao longo do tempo, em três momentos diferentes.

**Métodos:**

Estudo descritivo retrospectivo comparativo observacional dos resultados cirúrgicos obtidos pelo Serviço de Cirurgia Oncológica do Hospital Erasto Gaertner. A amostra foi dividida em: Período 1 (1987-1997), Período 2 (1998-2003) e Período 3 (2007-2015). Taxas de sobrevida e sobrevida livre de doença foram estimadas pelo método de Kaplan-Maier. Preditores de sobrevida foram identificados com regressão de Cox. Para a comparação entre os grupos foi utilizado teste ANOVA. Os dados foram analisados com os programas SPSS 25.0 e STATA 16, sendo p<0,05 considerado estatisticamente significativo.

**Resultados:**

Ao todo, 335 doentes foram submetidos a esofagectomia ou esofagogastrectomia. Quando comparadas as características clínicas dos 3 grupos não houve diferença estatística significativa. A realização de neoadjuvância foi significativamente maior no Período 3 (55,4% dos pacientes). Verificamos uma mudança histológica do diagnóstico no decorrer do tempo, com um aumento significativo do adenocarcinoma. As taxas de morbimortalidade foram superiores no Período 3. As principais complicações foram pulmonares e de fistulas anastomóticas. A sobrevida global em 5 anos foi aumentando no decorrer do tempo, atingindo 59,7% no Período 3.

**Conclusões:**

Melhor tratamento neoadjuvante contribuiu para aumentar a sobrevida global dos pacientes, apesar de maior incidência de complicações imediatas à cirurgia.

## INTRODUCTION

In Brazil, the National Institute of Cancer (INCA) estimates the diagnosis of 10,810 cases of esophageal cancer for the year 2016, 7,950 in men and 2,860 in women^1^. It represents the sixth most common cancer among men in the country, with remarkably close incidence and mortality rates^1^. The overall five-year survival is only between 15% and 25%^2^. Worldwide, the incidence of esophageal cancer varies according to the socio-economic-cultural level of the populations studied^3^. The highest rates are found in Southern and Eastern Africa, the Middle East, and East Asia, the region being known as The Esophageal Cancer Belt, with incidences between 15 and 22 cases per 100,000 men and 6.4 to 11.7 cases per 100,000 women in population age-adjusted rates. Meanwhile, in North and South America the incidence is close to 6/100,000 men and 2.1/100,000 women^3^.

 Among the gastrointestinal tract tumors, esophageal cancer has the particularity of presenting two predominant histological patterns: squamous cell carcinoma (SCC) and adenocarcinoma^4^. The esophagus is internally lined by squamous epithelium, from which the squamous cell carcinoma originates. In the distal third of the esophagus, secondary to chronic esophageal lesions, the squamous epithelium can differentiate into columnar intestinal mucosa, the tissue from which adenocarcinoma will originate^5^.

Squamous cell carcinoma represented more than 90% of cases until 20 years ago. However, the incidence of esophageal adenocarcinoma is growing and represents a considerable number of cases nowadays^5^ ^-^ ^7^. Recent studies highlight the different histological types according to incidence, risk factors, and outcomes^5^ ^-^ ^7^. Esophagogastric transition (EGT) adenocarcinomas have been described as tumors with the center 5.0 cm proximal or distal to the cardia^8^ ^-^ ^10^. Siewert et al. described three different tumor entities within the EGT: a) type I: the adenocarcinoma of the distal esophagus, which usually originates from an area of specialized intestinal metaplasia of the esophagus, that is, from a Barrett’s esophagus, and can infiltrate the localized transition distally; b) type II: true carcinoma of the cardia, which originates from the cardiac epithelium or short segments of intestinal metaplasia in the esophagogastric transition; c) type III: sub-cardiac gastric carcinoma, which infiltrates the proximally located esophagogastric transition^8^ ^-^ ^10^.

The standard treatment of esophageal cancer is still discussed in the literature, but the relevance of surgery is evident^11^. In all tumors considered resectable, surgery must be strongly considered. The candidates for esophagectomy are patients with tumors that invade the muscularis mucosae (T1b - T4a) without distant metastases at the time of diagnosis (M0)^12^. The current medical literature indicates combination therapy (surgery associated with chemotherapy and radiotherapy) to increase the control of the disease compared with surgery alone^13^ ^,^ ^14^. Patients who are candidates for the procedure should be transferred to reference centers with a large annual volume of surgeries^15^ ^,^ ^16^.

The trans-thoracic esophagectomy is the standard procedure performed around the world for treating esophageal neoplastic lesions^17^. Among the most used techniques there is the Ivor Lewis esophagectomy, performed with laparotomy associated with right thoracotomy^18^. This technique provides greater visualization of the intrathoracic esophagus, allowing for better dissection and greater margins, as well as a more comprehensive lymphadenectomy. This approach, however, is associated with greater perioperative cardiorespiratory impairment and a high risk of mediastinitis due to anastomotic fistula, which may progress to sepsis and death^17^ ^,^ ^18^. A three-incision approach (abdominal, thoracic and cervical) allows good dissection and the anastomosis at the cervical site, reducing the risk of mediastinitis^19^ ^,^ ^20^. With the evolution of radiotherapy techniques and chemotherapy drugs, the treatment of esophageal cancer has been modified over the years^17^.

This study aims to present the clinical and surgical data of patients with esophageal cancer operated in the same institution in three different historical moments, comparing the outcomes with the treatment strategies employed.

## METHODS

We conducted a retrospective, comparative, observational study. We included all patients who underwent surgical treatment for esophageal cancer at the Hospital Erasto Gaertner, in Curitiba-PR, Brazil, in three distinct chronological moments. The project was approved by the Ethics in Research Committee under number 1,122,319/2015.

Surgical indications: Patients with SCC affecting the middle third of the esophagus underwent esophagectomy. Patients with SCC or adenocarcinoma of the distal esophagus or of the cardiac region underwent esophagogastrectomy. When indicated, patients underwent neoadjuvance with chemo-radiotherapy and were operated between eight and 12 weeks after the end of radiotherapy. Regional lymphadenectomy was performed in all cases.

Diagnostic and staging routine: All patients underwent preoperative cardiovascular, pulmonary, renal, hepatic, nutritional, and anesthetic assessments, which might vary depending on the underlying disease. The presence of a recent imaging exam (less than 45 days) was mandatory for the procedure.

Variables collected: We collected and analyzed data on patients’ clinical characteristics, such as personal and family history, smoking and alcohol history, Performance-Status (PS), laboratory tests, tumor characteristics, associated treatments, transfusion requirements, length of stay, mortality, and postoperative complications according to the Clavien-Dindo classification.

Characteristics of the procedure: The surgical access route was conventional (open) in all cases. The procedure was always performed by the same medical team at the three moments. The Service Routine comprehends a three-field esophagectomy, with cervicotomy, right thoracotomy, and laparotomy in cases of tumors that involve the middle third of the esophagus and require cervical anastomosis. For tumors that affect the distal third, esophagogastrectomy is performed in two fields, with right lateral thoracotomy and median laparotomy, and intra-thoracic anastomosis. We leave cervical peri-anastomotic drains (when performed in three fields) and pleural drains. We do not carry out transhiatal esophagectomy. Patients were referred to the hospital’s Intensive Care Unit (ICU) for immediate postoperative care.

Separation of groups: We divided patients into groups according to the period in which the surgical treatment was performed: Period 1 - 1987 to 1997^21^; Period 2 - 1998 to 2003^22^; and Period 3 - 2007 to 2015. We considered a convenience sample, with no sample size calculation applicable (100% of the operated cases included).

Statistical analysis: We expressed data as mean and standard deviation or as median and interquartile range for non-normal distribution. We analyzed quantitative numerical variables with the ANOVA test. We used the Mann-Whitney U non-parametric test for numerical variables with non-normal distribution. We analyzed categorical variables using the Chi-square test with Fisher’s correction. We used the SPSS 23.0 and STATA 15 softwares, with p < 0.05 considered statistically significant.

Survival analysis: We carried out a survival analysis with univariate comparison. The factors considered in this analysis were local invasion (T3/T4), positive lymph nodes (N1), degree of differentiation, compromised margins, histological type, and neoadjuvant chemotherapy.

## RESULTS

In total, 335 patients underwent surgery, 132 in Period 1, 111 in Period 2, and 92 patients in Period 3. Males represented 71.2% in Period 1, 72.8% in Period 2, and 73.9% in Period 3, p = 0.34. The median age at diagnosis was 53.5 years in Period 1, 55 in period 2, and 60.8 in Period 3, p = 0.24. With respect to patients’ Performance Status, ECOG PS1 predominated (65.1%) in Period 1, ECOG-PS analysis was not performed in Period 2, and ECOG-PS1 preponderated (60.9%) in Period 3, p = 0.44. Table 1 shows the clinical-epidemiological data.

**Table 1:** Demographic characteristics and epidemiological profile of the sample of 335 patients with esophageal cancer undergoing surgery.

Variable	Period 1 (n = 132)	Period 2 (n = 111)	Period 3 (n = 92)	p value
Sex, male (%)	94 (71.2)	82 (72.8)	68 (73.9)	0.34
Age, years (median, IQR)	53,5	55	60.8 (54.3-65.5)	0.24
ECOG PS, number (%)				0.44
0	4 (3)	NE	21 (22.8)	
1	86 (65.1)	NE	56 (60.9)	
2	42 (31.9)	NE	12 (13)	-
BMI (kg/m2), (IQR)	NE	NE	22.9 (19.9-25.9)	-
Smoking, number (%)	88 (66.7)	NE	72 (78.3)	-
Smoking, pack/years (IQR)	NE	NE	24.50 (5.00-40.00)	-
Alcoholism, number (%)	57 (43.2)	NE	45 (54.9)	-
Hypertension and heart disease, number (%)	20 (15.1)	NE	24 (26.1)	-
Family history of cancer, number (%)	12 (9.1)	NE	31 (43.7)	-
Personal history of cancer, number (%)	NE	NE	10 (10.9)	-

IQR - interquartile range; ECOG-PS - Eastern Cooperative Oncology Group - Performance Status; BMI - Body Mass Index; NE - not evaluated.

The histological type was adenocarcinoma in 4.5% of patients in Periods 1 and 2, and in 15.2% in Period 3, p < 0.001. There was lymph node involvement in 54.5% in Period 1, in 22.7% in Period 2, and in 46.2% in Period 3, p = 0.64.

Neoadjuvance (chemo radiotherapy) was employed in 29.5% in Period 1, in 12.6% in Period 2, and in 52.2% in Period 3, p < 0.001. Partial or complete response to neoadjuvance was not analyzed in periods 1 and 2, and was 72.9% in Period 3.

The median follow-up was 2.5 years for Period 1, 1.8 years for Period 2, and 1.9 years for Period 3, p < 0.001. Mortality was 17.3% in Period 1, 9% in Period 2, and 37% in Period 3, p < 0.001. The recurrence rate was 45 (34.1%) for Period 1, 32 (28.8%) for Period 2, and 31 (33.7%) for Period 3, p < 0.001. Table 2 shows data related to tumor characteristics, neoadjuvant therapy, and follow-up.

**Table 2:** Histopathological and oncological characteristics of the 335 patients with esophageal cancer undergoing surgery.

Variable	Period 1 (n = 132)	Period 2 (n = 111)	Period 3 (n = 92)	p value
Histological type, number (%)				<0.001
Adenocarcinoma	6 (4.5)	5 (4.5)	14 (15.2)	
SCC	125 (94.7)	106 (95.5)	73 (79.3)	
Others	1 (0.8)	0 (0)	5 (5.4)	
Degree of differentiation, number (%)				-
Well differentiated	12.00 (9.1)	NE	17 (20)	
Moderately differentiated.	77 (58.3)	NE	57 (67.1)	
Poorly differentiated/ Undifferentiated	28 (21.2)	NE	11 (12.9)	
Local invasion, number (%)				0.54
T1a	-	-	6 (6.8)	
T1b	43 (32.6)	6 (5.4)	6 (6.8)	
T2	58 (43.9)	16 (14.4)	25 (28.4)	
T3	12 (9.1)	82 (73.9)	45 (51.1)	
T4a	4 (3.1)	7 (6.3)	4 (4.5)	
T4b	-	-	2 (2.3)	
Free margins, number (%)	132 (100)	109 (98.2)	77 (83.7)	<0.001
Lymph node involvement, number (%)	72 (54.5)	25 (22.7)	42 (46,2)	0.64
Neoadjuvancy, number (%)	39 (29.5)	14 (12.6)	48 (52.2)	<0.001
Neoadjuvant chemotherapy, number of cycles (IQR)	2.4 (1-6)	NE	3 (2-6)	-
Neoadjuvant radiation therapy, dose, Grays (IQR)	46 (20-65)	NE	47 (38-50)	-
Response to neoadjuvancy, number (%)				-
Complete response	NE	NE	12 (25)	
Partial response	NE	NE	23 (47.9)	
Stable disease	NE	NE	13 (27.1)	
Disease progression	NE	NE	-	
Recurrence rate, number (%)	45 (34.1)	32 (28.8)	31 (33.7)	<0,001
Mortality, number (%)	18 (17.3)	10 (9)	34 (37)	<0,001
Median follow-up, years (IQR)	2.5	1.8	1.9 (0.1-5.9)	<0,001

IQR - interquartile range; SCC - squamous cell carcinoma; NE - not evaluated.

As for the surgical variables in the three historical moments, in Period 3 there was a shorter hospital stay, a smaller number of dissected lymph nodes, and a higher rate of postoperative fistulas. In addition, morbidity and mortality were higher in Period 3. The main complications were pulmonary ones and anastomotic fistulas. Table 3 shows the surgical data.

**Table 3:** Variables related to the surgical procedure in the 335 patients with esophageal cancer undergoing surgery.

Variable	Period 1 (n = 132)	Period 2 (n = 111)	Period 3 (n = 92)	p value
ASA, number (%)				-
0	NE	NE	16 (17.4)	
1	NE	NE	64 (69,.6)	
2	NE	NE	11 (12)	
Preoperative hemoglobin, mg/dl (IQR)	NE	NE	13.7 (12.4-14.7)	-
Preoperative leukocytes, number (IQR)	NE	NE	8055 (6190-10025)	-
Blood transfusion, number (%)	NE	NE	19 (20.7)	-
Red blood cell concentrate, units (IQR)	1,7 (0-3.6)	NE	2 (1-4)	-
Surgical time, minutes, median (IQR)	343	NE	360 (300-390)	0.24
Dissected lymph nodes, number (IQR)	17 (3-50)	22.6 (4-50)	12 (7.2-17.7)	<0.001
Length of stay, days, median (IQR)	15	NE	10 (8-15)	<0.001
ICU time, days, median (IQR)	NE	NE	5 (3-6)	-
Clavien-Dindo 3 4 5 postoperative complication, number (%)	52 (39.4)	44 (39.6)	37 (40.2)	0.04
Fistula, number (%)	9 (6.8)	7 (6.3)	19 (20.7)	<0.001
Reoperation, number (%)	NE	NE	19 (20.7)	-

ASA - American Society of Anesthesiology; IQR - interquartile range ; ICU - Intensive Care Unit; NE - not evaluated.

The rate of transfused patients was 20.7% in Period 3. The median operative time was 343 minutes in the Period 1, not recorded in Period 2, and 360 min in Period 3, p = 0.54. Of all patients, 39.4% had Clavien-Dindo 3-4-5 postoperative complications in Period 1, 36.6% in Period 2, and 40.2% in Period 3, p = 0.64. Of these, 20.7% were reoperated on Period 3. The median hospitalization time was 15 days in Period 1, was not measured in Period 2, and was 10 days in Period 3, p < 0.001. The fistula rate was 6.8% in Period 1, 6.3% in Period 2, and 20.7% in Period 3, p < 0.001. The main postoperative complications were anastomotic fistula, pneumonia, pneumothorax, sepsis, chylothorax, and stroke.

**Figure 1:**
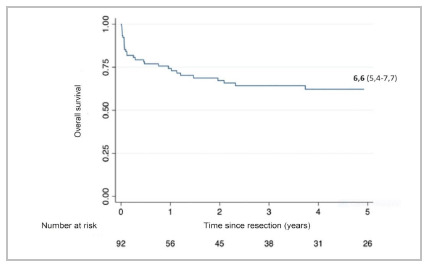
Overall and median survival in years of the 92 patients with esophageal cancer undergoing surgery in Period 3.

As for cancer results, the estimated overall survival at one, three, and five years was 59.8%, 40.2%, and 27.2%, respectively, in Period 3. The overall one-year survival rate was 45.5% in Period 1, 48.4% in Period 2, and 59.8% in Period 3, p = 0.02. The median overall survival was 1.8 years in Period 1, 3 years in Period 2, and 6.6 years in Period 3, p = 0.02. Figure 1 shows the median overall survival curve of Period 3.

The univariate Cox regression for disease-free survival showed that the increased number of recurrences were associated with local invasion greater than T2 [HR 2.3 (95% CI 1.1 4.9)], positive lymph nodes [HR 3.4 (95% CI 1.6 7.1)], degree of tumor differentiation [HR 7.1 (95% CI 0.9 51.8)], and neoadjuvant chemotherapy [HR 2.9 (95% CI 1.4 6.2)]. Table 4 shows the Cox regression results.

**Table 4:** Univariable analysis with Cox regression for disease-free survival of the 335 patients with esophageal cancer undergoing surgery.

Variable	Univariate analysis	
	HR CI 95%	p value
Local invasion (ref.: T1 / T2)	2.3 (1.1-4.9)	0.02
Positive lymph nodes (ref.: N0)	3.4 (1.6-7.1)	<0.01
Degree of differentiation	7.1 (0.9-51.8)	0.05
(ref.: well-differentiated)		
Free margins (ref.: yes)	0.9 (0.4-2.4)	0.89
Histological type	1.4 (0.5-4.1)	0.51
(ref.: adenocarcinoma)		
Neoadjuvant chemotherapy (ref.: no)	2.9 (1.4-6.2)	<0.01

HR - hazards ratio; CI - confidence interval; T - tumor size staging; N - lymph node staging.

## DISCUSSION

This study shows a change of histology diagnosed during recent decades, with a significant increase in the incidence of adenocarcinoma. Imamura et al. demonstrated the same pattern of etiological behavior in esophageal cancer in Japan^23^.

Although there was no difference with statistical significance, there was a trend of increased cancer specific survival over the years. The wide diffusion of chemo-radiotherapy in different countries as neoadjuvant therapy for esophageal cancer is making treatment increasingly successful^24^ ^,^ ^25^. Similarly, the increase in the indication of neoadjuvancy for the treatment of esophageal cancer is related to better survival rates, despite higher rates of serious postoperative complications. The study by Bang et al. corroborates this, showing higher rates of postoperative complications and increased anastomotic leaks in 12% of patients submitted to neoadjuvant therapy^26^.

Patients in Period 3 were older and with higher ECOG-PS compared with individuals treated in the other periods, which may have contributed to the higher absolute mortality in this population, followed-up for a longer time after operation^27^. There was a higher rate of fistulas in Period 3, related to the more frequent preoperative radiotherapy, which renders the irradiated tissues more fragile. Similarly, after irradiation a significant part of the lymph nodes undergoes apoptosis, being more difficult to detect in postoperative histopathological analyzes, underestimating the lymphadenectomy performed. Van den Ende et al. found an ECOG-PS greater than 1 to be related with higher rates of surgical complications^24^. In addition, more modern radiotherapy techniques associated with better chemotherapy drugs for neoadjuvancy have led to the expansion of criteria for the indication of surgery and the limits of cancer resection, which contributes to higher rates of postoperative complications. On the other hand, patients with tumors with less aggressive biological behavior end up being selected during neoadjuvant treatment to not undergo surgery^24^. De Gouw et al. demonstrated that the response to neoadjuvant treatment appears as one of the main predictors of tumor biological behavior^28^. 

Even with all advances in treatment, the importance of early diagnosis is clear in esophageal cancer. Prevention and early diagnosis strategies are major steps at increasing cancer specific and overall survival in different populations and should be the main focus of current cancer treatment^29^. The Cox regression demonstrated the impact of tumor size, as well as lymph node spread and the degree of differentiation, for oncological results of tumor recurrence. These factors also appear as the most important predictors of recurrence over five years of follow-up.

The present study is a comparison of three surveys of clinical-surgical oncological results carried out independently over the past decades at the institution. For this reason, there is an important limitation in the effective comparison of all variables’ clinical applicability, since there was no pattern of data collection from the patients’ medical records at the three different historical moments. However, technically possible comparisons were made with existing data.

## CONCLUSION

Better neoadjuvant treatment contributed to increase the overall survival of patients, despite a higher incidence of immediate postoperative complications.
